# Prevalence of left atrial enlargement and its risk factors in general Chinese population

**DOI:** 10.1186/s12872-016-0229-z

**Published:** 2016-03-05

**Authors:** Qiaoyun Ou, Yintao Chen, Shasha Yu, Xiaofan Guo, Huijie Zhao, Yingxian Sun

**Affiliations:** Department of Cardiology, The First Hospital of China Medical University, 155 Nanjing North Street, Heping District, Shenyang, 110001 China

**Keywords:** Left atrial enlargement, Prevalence, Risk factors, General Chinese population

## Abstract

**Background:**

Left atrial enlargement (LAE) has been proven to be significantly related to stroke and cardiovascular diseases. In China, few studies related to LAE have been conducted, especially in the general population. To the best of our knowledge, our study is the first to explore the prevalence of LAE and associated risk factors in the general Chinese population.

**Methods:**

This study included a total of 11,956 subjects aged ≥35 years. All participants completed a questionnaire, and underwent complete physical examination, electrocardiogram (ECG) and echocardiogram. We defined LAE as a LA diameter exceeding 4.0 cm in men and 3.8 cm in women. We conducted a multivariable logistic regression analysis and a linear regression analysis to identify independent factors of LAE.

**Results:**

The overall prevalence of LAE was 6.43 % for subjects aged over 35 years. The prevalence of LAE was 6.78 % in women and 6.02 % in men. The major risk factors of LAE were female sex (odds ration [OR]: 1.229, β: 0.197), advancing age (OR: 1.015, β: 0.036), high systolic blood pressure (OR: 2.331, β: 0.185), high body mass index (BMI) (OR: 3.956, β: 0.373), diabetes (OR: 1.498, β: 0.030), high left ventricular myocardial index (OR: 1.003, β: 0.073), and low left ventricular ejection fraction, low heart rate, and low estimated glomerular filtration rate. Additionally, the association between BMI and LAE was the most obvious.

**Conclusions:**

Female sex, advancing age, high systolic blood pressure, high BMI, diabetes, high left ventricular myocardial index, low estimated glomerular filtration rate, low left ventricular ejection fraction, and low heart rate were proven to be risk factors of LAE.

## Background

Several studies have shown that alterations in the left ventricular structure and function are associated with an increased risk of cardiovascular morbid and fatal events [1–3]. Recently, left atrial enlargement (LAE) has been proven to be significantly related to stroke and cardiovascular diseases [4–7]. Furthermore, some studies have found that LAE may be a more sensitive indicator of cardiovascular diseases, compared with left ventricular remodeling [8, 9]. Therefore, the study of the prevalence of LAE and its risk factors is becoming increasingly important. However, very few studies conducted on the general population have focused on LAE. At present, almost all of the research is aimed at patients with high blood pressure (BP), or those who are hospitalized. Moreover, no research studies on the general Chinese population have focused on LAE. To the best of our knowledge, our study is the first to explore the prevalence of LAE and its risk factors in the general Chinese population, including over 10,000 participants. The aims of our study were as follows: (1) to investigate the prevalence of LAE in a rural Chinese population consisting of a general adult population rather than patients that were indicated to undergo echocardiographic examination for different reasons, (2) and to explore independent risk factors of LAE.

## Methods

### Study population

From January 2012 to August 2013, a representative sample of participants aged ≥35 years was selected to characterize the prevalence and risk factors in rural areas of Liaoning Province, located in China. The study adopted a multi-stage, stratified, random-cluster sampling scheme. In the first stage, three counties (Dawa, Zhangwu and Liaoyang County) were randomly selected from Liaoning province. In the second stage, one town was randomly selected from each county (for a total of three towns). In the third stage, 8–10 rural villages from each town were randomly selected (for a total of 26 rural villages). Participants who were pregnant or had malignant tumors or mental disorders were excluded from the study, comprising a potential pool of 14,016 people. Of these, 11,956 participants agreed and completed the present study, yielding a response rate of 85.3 %. In this report, we used only the data from participants who completed the study, which provided a final sample size of 10,574 subjects (4768 men and 5806 women). For the present analysis, participants with atrial fibrillation showed by Electrocardiogram that made diastolic function and diameter assessment unreliable with conventional Doppler technique were also excluded.

The study was approved by the Ethics Committee of China Medical University (Shenyang, China). All procedures were performed in accordance with ethical standards. Written consent was obtained from all participants after they had been informed of the objectives, benefits, medical items and confidentiality agreement regarding their personal information. For participants who were illiterate, we obtained written informed consent from their proxies.

### Data collection and measurements

Data were collected during a single visit to the clinic by cardiologists and trained nurses using a standard questionnaire in a face-to-face interview. Before the survey was performed, we invited all eligible investigators to attend an organized training session. The training included the purpose of this study, how to administer the questionnaire, the standard method of measurement, the importance of standardization and the study procedures. A strict test was administered after this training, and only those who scored perfectly on the test were accepted as investigators in this study. During data collection, our inspectors gave some further instructions and support. Data regarding the demographic characteristics, lifestyle risk factors, dietary habits, family income and family history of chronic diseases were obtained during the interview using the standardized questionnaire. The study was guided by a central steering committee with a subcommittee for quality control.

According to American Heart Association protocol, BP was measured three times at 2-min intervals after at least 5 min of rest using a standardized automatic electronic sphygmomanometer (HEM-907; Omron), which had been validated according to the British Hypertension Society protocol [10] . The participants were advised to avoid caffeinated beverages and exercise for at least 30 min before the measurement. During the measurement, the participants were seated with their arms supported at the level of the heart. The mean of three BP measurements was calculated and used in all analyses. Weight and height were measured to the nearest 0.1 kg and 0.1 cm, respectively, with the participants wearing light-weight clothing and without shoes. Body mass index (BMI) was calculated as the weight in kilograms divided by the square root of the height in meters. Fasting blood samples were collected in the morning after at least 12 h of fasting. Blood samples were obtained from an antecubital vein into Vacutainer tubes containing ethylenediaminetetraacetic acid (EDTA). Fasting plasma glucose (FPG), total cholesterol (TC), low-density lipoprotein cholesterol (LDL-C), high-density lipoprotein cholesterol (HDL-C), triglycerides (TGs) and other routine blood biochemical indexes were analyzed enzymatically using an autoanalyzer. All laboratory equipments were calibrated, and blinded duplicate samples were used for these analyses.

### Echocardiographic measurements

Echocardiograms were obtained using a commercially available Doppler echocardiograph (Vivid, GE Healthcare, United States), with a 3.0-MHz transducer. The transthoracic echocardiogram examinations included M-mode, two-dimensional, spectral and color Doppler, and were performed with subjects resting in the supine position. Echocardiogram analyses and readings were performed by three doctors specialized in echocardiography. Consultations were made to two other specialists if any questions or uncertainty arose. The parasternal acoustic window was used to record two-dimensional and M-mode images of the left ventricular (LV) internal diameter, wall thickness, aortic root and left atrium (LA). The apical acoustic window was used to record 4- and 5-chamber images. Color Doppler recordings were adopted to identify valvular regurgitation. The correct orientation of imaging planes and Doppler recordings were verified using previously described procedures [11, 12]. The left ventricular end-diastolic dimension (LVIDd) was obtained in the LV minor axis at end-diastole, internal dimensions and interventricular septal thickness (IVST) and posterior wall thickness (PWT) were measured at end of the diastole and systole according to the recommendations of the American Society of Echocardiography [13, 14]. The LV mass was estimated by Devereux’s formula 0.8 × [1.04 {(LVIDd + PWT+ SWT)^3^ − LVIDd^3^}] + 0.6 g [15] and normalized to body surface area (BSA). Left ventricular ejection fraction (LVEF) was measured from the four-chamber apical projection by the area product × the ventricular length. Two-dimensional guided M-mode measurements of the LA posteroanterior dimension were performed from the parasternal long-axis view according to the standards of the American Society of Echocardiography.

### Definitions

Left atrial enlargement was defined as an LA diameter exceeding 4.0 cm in men and 3.8 cm in women [16]. LAE as the index by body surface area (iLAE) was defined as indexed LA diameter exceeding 2.3 cm/m2 in both sexes [16]. Higher left ventricular myocardial index (LVMI) was defined as LVMI exceeding 115 g/m^2^ in men and 95 g/m^2^ in women [16]. According JNC-7 report [17], Hypertension was defined as systolic blood pressure (SBP) ≥140 mmHg and/or diastolic blood pressure (DBP) ≥90 mmHg and/or use of antihypertensive medications. BMI were categorized into 2 groups as normal (BMI <30 kg/m^2^) and obesity (BMI ≥30 kg/m^2^), according to the World Health Organization (WHO) criteria [18]. Dyslipidemia was defined according to the National Cholesterol Education Program-Third Adult Treatment Panel (ATP III) criteria [19]. Diabetes mellitus was diagnosed according to the WHO criteria [20]: FPG ≥ 7 mmol/L (126 mg/dL) and/or being on treatment for diabetes. Anemia was defined as an hemoglobin concentration lower than 110 g/L in women and lower than 120 g/L in men according to the China expert consensus.

### Statistical analysis

Descriptive statistics were calculated for all the variables, including continuous variables (reported as mean values and standard deviations) and categorical variables (reported as numbers and percentages). The differences between the LAE and non-LAE groups were evaluated using the Student’s *t*-test, analysis of variance, non-parametric test or the *χ*^2^**-**test, as appropriate. Multivariate logistic regression analyses and linear regression analysis were used to identify independent factors of LAE, and odds ratios (ORs). Linear correlation coefficient β and corresponding 95 % confidence intervals (CIs) also were calculated. All the statistical analyses were performed using SPSS version 17.0 software (SPSS Inc, Chicago, Illinois, USA), and *P* values less than 0.05 were considered statistically significant.

## Results

### Background characteristics

Patient baseline characteristics are shown in Table 1. A total of 10,574 participants (4768 men and 5806 women) were included in the study. The mean age was 53.81 years. The subjects in the LAE group were older and included a higher proportion of women than the non-LAE group (*P* < 0.001 for both). Participants with LAE had higher BP, higher body mass index (BMI), higher prevalence of diabetes mellitus, higher TG, higher TC, higher LVMI, lower left ventricular ejection fraction, and lower estimated glomerular filtration rate (eGFR) than those in the non-LAE group (*P* < 0.001).

**Table 1:** Characteristics of the study population

Characteristics	Total	LAE	Non-LAE	*P*
Gender, female	5991 (54.16 %)	406 (57.10 %)	5585 (53.96 %)	0.104
Age, y	53.81 ± 10.52	57.86 ± 10.40	53.53 ± 10.47	<0.001
currentsmoking	3890 (35.17 %)	194 (27.29 %)	3696 (35.71 %)	<0.001
Currentdrinking	2476 (22.39 %)	151 (21.24 %)	2325 (22.46 %)	0.448
SBP, mmHg	141.63 ± 23.36	158.18 ± 25.64	140.5 ± 22.76	<0.001
DBP, mmHg	81.97 ± 11.70	86.87 ± 12.52	81.63 ± 11.49	<0.001
HR, beats minute	71.66 ± 12.3	71.03 ± 11.90	71.70 ± 12.33	0.165
BMI, Kg/m^2^	24.78 ± 3.66	27.54 ± 3.77	24.60 ± 3.58	<0.001
Diabetes mellitus	1148 (10.38 %)	133 (18.71 %)	1015 (9.81 %)	<0.001
HDL-C, mol/L	1.41 ± 0.38	1.32 ± 0.32	1.41 ± 0.38	<0.001
LDL-C, mol/L	2.93 ± 0.82	3.08 ± 0.85	2.92 ± 0.82	<0.001
TG, mmol/L	1.63 ± 1.47	1.89 ± 1.42	1.62 ± 1.48	<0.001
TC, mmol/L	5.24 ± 1.09	5.38 ± 1.11	5.23 ± 1.08	<0.001
eGFR, mL/min/1.73 m^2^	92.81 ± 15.87	88.02 ± 18.51	93.14 ± 15.62	<0.001
Hemoglobin, g/L	138.6 ± 18.74	138.28 ± 24.50	138.63 ± 18.29	0.638
LVDd, cm	4.71 ± 0.43	5.07 ± 0.55	4.68 ± 0.41	<0.001
IVST, cm	0.89 ± 0.26	1.01 ± 0.38	0.88 ± 0.24	<0.001
PWT, cm	0.87 ± 0.26	0.96 ± 0.36	0.86 ± 0.25	<0.001
LVMI, g/m^2^	86.83 ± 59.31	110.44 ± 87.80	85.21 ± 56.48	<0.001
LVEF, %	62.97 ± 3.81	61.33 ± 4.77	63.09 ± 3.71	<0.001
E/A	1.06 ± 4.28	0.91 ± 0.74	1.07 ± 4.42	0.339

*Abbreviations*: *LAE* left atrial enlargement, *Non-LAE* non-left atrial enlargement, *BMI* body mass index, *SBP* systolic blood pressure, *DBP* diastolic blood pressure, *TC* total cholesterol, *TG* triglycerides, *LDL-C* low density lipoprotein cholesterol, *HDL-C* high density lipoprotein cholesterol, *eGFR* glomerular filtration rate, *HR* heart rate, *LVDd* left ventricular end-diastolic dimension, *IVST* interventricular septal thickness, *PWT* posterior wall thickness, *LVEF* left ventricular ejection fraction

Note: data are expressed as mean ± standard deviation or *n* (%)

### Prevalence of LAE in different sexes and ages

**Fig. 1:**
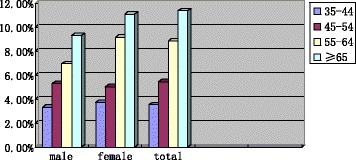
Prevalence of left atrial enlargement in different gender and age

The overall prevalence of LAE was 6.43 % for subjects aged over 35 years. The prevalence of LAE was 6.78 % in women and 6.02 % in men. For men, the prevalence of LAE increased with advancing age (age 35–44 years: 3.32 %; age 45–54 years: 5.31 %; age 55–64 years: 6.95 %; and age ≥65 years: 9.31 %). For women, the prevalence of LAE was 3.72, 5.03, 9.17, 11.11 %, respectively. As for the total population, the prevalence was 3.55, 5.44, 8.85, 11.37 % respectively (Fig. 1).

After indexed by body surface area, the overall prevalence of iLAE was 15.35 % for subjects aged over 35. The prevalence of iLAE was 20.58 % in female, 9.09 % in male. For men, the prevalence of LAE increased with advancing age (age 35–44: 2.3 %; age 45–54: 5.5 %; age 55–64: 11.7 %; age ≥ 65: 20.3 %). As for women, the prevalence was 9.2, 14.2, 25.6, 44.8 %. As for whole population, the prevalence was 6.2, 10.3, 19.1, 32.6 %.

### Factors associated with LAE

Table 2 shows multivariable logistic regression analysis of risk factors related to LAE. The prevalence of LAE was higher for women than men (OR: 1.229, 95 % CI: 1.003–1.507). Advancing age (OR: 1.015, 95 % CI: 1.005–1.026), high systolic blood pressure (SBP) (OR: 2.331, 95 % CI: 1.889–2.876), high diastolic blood pressure (DBP) (OR: 1.24, 95 % CI: 1.016–1.513), high BMI (OR: 3.956, 95 % CI: 3.208–4.879), high prevalence of diabetes (OR: 1.498, 95 % CI: 1.19–1.886), high LVMI (OR: 1.003, 95 % CI: 1.002–1.004), and lower LVEF, lower heart rate, and lower eGFR were found to be the major risk factors of LAE. After discussing the association between men and women, respectively, no obvious correlation was found between DBP and LAE. The associations between diabetes, eGFR and LAE were significant in men (*P* < 0.05 vs. *P* < 0.05), but not in women (*P* = 0.263 vs. *P* = 0.751). Additionally, we tested the association by linear regression analysis (Table 3). Female sex, advancing age, high SBP, high BMI, high fasting plasma glucose, high LVMI, low eGFR, low LVEF, and low heart rate were proven to be highly significant risk factors of LAE. Additionally, the association between BMI and LAE was shown to be the most obvious.

**Table 2:** Multivariable logistic regression analysis of risk factors related to left atrial enlargement

Characteristics	Total		Male		Female	
	OR (95 % CI)	*P*	OR (95 % CI)	*P*	OR (95 % CI)	*P*
Gender, female	1.229 (1.003–1.507)	0.046				
Age	1.015 (1.005–1.026)	0.005	1.007 (0.992–1.022)	0.383	1.017 (1.002–1.032)	0.026
Current smoking	0.741 (0.607–0.905)	0.003	0.741 (0.570–0.963)	0.025	0.730 (0.533–0.998)	0.049
Current drinking	1.274 (1.001–1.62)	0.048	1.291 (0.987–1.688)	0.063	1.308 (0.715–2.393)	0.384
SBP ≥ 140	2.331 (1.889–2.876)	0.000	2.673 (1.901–3.758)	0.000	2.033 (1.551–2.664)	0.000
DBP ≥ 80	1.24 (1.016–1.513)	0.034	1.302 (0.966–1.754)	0.083	1.161 (0.888–1.518)	0.276
HR	0.980 (0.972–0.987)	0.000	0.979 (0.967–0.990)	0.0002	0.978 (0.968–0.988)	0.000
BMI ≥ 30	3.956 (3.208–4.879)	0.000	4.947 (3.53–6.932)	0.000	3.414 (2.61–4.467)	0.000
Diabetes mellitus	1.498 (1.19–1.886)	0.0005	2.042 (1.443–2.889)	0.000	1.192 (0.877–1.518)	0.263
TC	0.953 (0.880–1.033)	0.242	0.977 (0.860–1.11)	0.720	0.931 (0.839–1.033)	0.176
TG	1.029 (0.977–1.083)	0.277	0.973 (0.899–1.054)	0.507	1.076 (1.002–1.154)	0.043
eGFR,	0.991 (0.985–0.997)	0.004	0.979 (0.969–0.989)	0.000	0.999 (0.991–1.007)	0.751
Anemia	1.253 (0.981–1.6)	0.07	1.222 (0.771–1.938)	0.394	1.326 (0.990–1.774)	0.058
LVMI	1.003 (1.002–1.004)	0.000	1.002 (1.000–1.003)	0.009	1.004 (1.003–1.006)	0.000
LVEF	0.928 (0.911–0.946)	0.000	0.939 (0.913–0.966)	0.000	0.919 (0.896–0.942)	0.000
E/A	0.892 (0.703–1.133)	0.350	1.017 (0.934–1.107)	0.695	0.523 (0.348–0.788)	0.002

**Table 3:** Linear regression analysis of risk factors related to left atrial enlargement

Characteristics	Total		Male		Female	
	β (95 % CI)	*P*	β (95 % CI)	*P*	β (95 % CI)	*P*
Gender, female	0.197 (−0.174, −0.139)	0.000				
Age	0.036 (0.0005, 0.002)	0.002	0.017 (−0.0006, 0.002)	0.335	0.049 (0.0005, 0.003)	0.006
Current smoking	0.001 (−0.015, 0.016)	0.897	0.001 (−0.020, 0.021)	0.945	−0.002 (−0.027, 0.023)	0.873
Current drinking	0.030 (0.009, 0.047)	0.003	0.037 (0.008, 0.049)	0.006	−0.001 (−0.056, 0.052)	0.951
Mean SBP	0.185 (0.003, 0.004)	0.185	0.176 (0.002, 0.003)	0.000	0.194 (0.003, 0.004)	0.000
Mean DBP	−0.045 (−0.002, −0.0006)	0.034	−0.018 (−0.002, 0.001)	0.373	−0.066 (−0.003, −0.001)	0.0002
HR	−0.122 (−0.005, −0.003)	0.000	−0.138 (−0.005, −0.004)	0.000	−0.119 (−0.005, −0.003)	0.000
BMI	0.373 (0.038, 0.042)	0.000	0.380 (0.038, 0.044)	0.000	0.378 (0.037, 0.042)	0.000
FPG	0.030 (0.003, 0.012)	0.0005	0.037 (0.002, 0.015)	0.000	0.026 (0.0004, 0.012)	0.263
TC	−0.019 (−0.014, −0.0004)	0.038	−0.007 (−0.012, −0.008)	0.641	−0.036 (−0.021, −0.003)	0.006
TG	0.014 (−0.001, 0.008)	0.146	−0.011 (−0.009, −0.004)	0.434	0.032 (0.002, 0.017)	0.012
eGFR	−0.029 (−0.001, −0.0002)	0.005	−0.050 (−0.002, −0.0005)	0.001	−0.009 (−0.0009, 0.0005)	0.540
Hemoglobin	−0.059 (−0.002, −0.0008)	0.000	−0.038 (−0.001, −0.0002)	0.005	1.326 (0.990–1.774)	0.058
LVMI	0.073 (0.0004, 0.0007)	0.000	0.075 (0.0003, 0.0006)	0.000	0.076 (0.0005, 0.0009)	0.000
LVEF	−0.038 (−0.006, −0.002)	0.000	−0.013 (−0.004, 0.001)	0.332	−0.059 (−0.008, −0.004)	0.000
E/A	−0.007 (−0.009, 0.003)	0.392	−0.002 (−0.007, 0.006)	0.897	−0.039 (−0.063, −0.011)	0.005

## Discussion

Our study found that the prevalence of LAE was 6.43 % in the total population, 6.78 % in women, and 6.02 % in men. A survey found that the prevalence of LAE defined according to LA diameter in an urban population in Poland was 15.7 %. As for the indexed LA diameter, the prevalence was 8.8 % [22]. It was found that their participants were much older (mean age of 63.0 years) and obese (mean BMI of 27.9 kg/m^2^), which may be the reason for the higher LAE prevalence among them than in our population. Stritzke et al. reported that the prevalence of LAE consists of 9.8 % the German residents [23]. However, they defined LAE as iLA (left atrial volume indexed in relation to body height) ≥35.7 and ≥33.7 ml/m in men and women, respectively. Thus, we suspect that the differences in prevalence between their study and ours can be attributed to the different population studied, different age levels, different BMI levels, different BP levels, and different definitions of LAE. Further, compared to Europeans and Americans, Asians tend to have smaller, thinner bodies. Furthermore, our research population was much larger. Thus, it is understandable that the prevalence of LAE in our study was relatively low.

In addition, we also found that the prevalence of LAE as the index by BSA was higher than that defined by absolute LA diameter, especially among the women population. After indexed by body surface area, the prevalence of iLAE was 15.35 % in total population, 20.58 % in female, and 9.09 % in male. This finding was different from some studies in Europe and America [22]. The reason for this difference is that Asians tend to have smaller, thinner bodies, compared to Europeans and Americans, especially in elder women. Their BSA was smaller, so when LAE was defined as LA diameter normalized to BSA *>*2.3 cm/m2 in both sexes, the prevalence of LAE in Asians became higher than Europeans and Americans, and the prevalence of iLAE was significantly higher in females than in males. A study in Japan confirmed the results to some extent [24]: Although the LA volume was similar between males and females, the LA volume indexed by BSA was significantly higher in females than in males.

Our study results indicated that advancing age and female sex were significantly associated with LAE, which has been reported in previous studies [9, 25]. In the case of advancing age, there are a few possible causes: valvular degeneration, cardiac systolic or diastolic dysfunction, hypertension among other factors. Compared to men, the higher prevalence of LAE in women may be ascribed to several factors, including higher systolic (143.4 vs. 139.9 mmHg) and mean arterial pressure (103.5 vs. 100.3 mmHg), and higher LVMI (91.85 vs. 81.77 g/m^2^), among other factors.

In our research, high SBP was significantly associated with LAE in different adjusted models. After adjusting for sex, DBP was not found to have an obvious correlation with LAE, which was consistent with many other studies [23]. Stritzke et al. found that LA pressure load in hypertensive individuals resulted in an increase in LA size [23]. Additionally, the association between BMI and LAE was shown to be the most obvious in our study, which was consistent with many other studies [9, 23]. Especially, Stritzke et al. reported that obesity appears to be the most important risk factor for LAE in the general population [23]. We also found an important result: diabetes and lower eGFR were positively associated with LAE in the general population, which was also consistent with previous studies [27, 28]. However, in our study, the association between diabetes, eGFR and LAE was only observed in men but not in women, after adjusting for sex.

Actually, previous studies confirmed that higher LVMI was associated with left atrial size [26, 27]. Our study further validates that affirmation. Appleton et al. showed that increased LVMI was related to increased LV stiffness and increase in LV filling pressure, which leads to LA enlargement [25]. Furthermore, our finding that heart rate was inversely related to LAE was consistent with previous studies [26, 28].

### Limitations

The present study had several limitations. First, our study was a cross-sectional study, which restricted the interpretation of the observed associations in terms of causality. Second, recent studies have shown that LA volume may be more accurate for the definition of LAE than LA diameter; therefore, the lack of LA volume data is a limitation of this study. However, the simple linear measurement is more common and convenient in daily clinical practice. Additionally, we consider that LA diameter is still helpful in identifying high-risk individuals.

## Conclusion

Our population-based study indicated that the latest prevalence of LAE in rural areas of China is not as high as that in European and American countries. The prevalence was similar between males and females, overall the women was slightly higher. After indexed by BSA, the prevalence of iLAE became higher, and it was significantly higher in females than in males. Female sex, advancing age, high SBP, higher BMI, diabetes, higher LVMI, lower eGFR, lower LVEF, and lower HR were found to be risk factors of LAE. Further prospective studies are required to verify these findings.

### Ethics approval and consent to participate

The study was approved by the Ethics Committee of China Medical University (Shenyang, China). All procedures were performed in accordance with ethical standards. Written consent was obtained from all participants after they had been informed of the objectives, benefits, medical items and confidentiality agreement regarding their personal information. For participants who were illiterate, we obtained written informed consent from their proxies.

### Consent for publication

Consent.

### Availability of data and materials

All of data and materials are availability.
